# Pain Management Strategies for Botulinum Toxin Type A Injections in Children: A Comprehensive National Survey and Procedural Protocol

**DOI:** 10.7759/cureus.48311

**Published:** 2023-11-05

**Authors:** Elisa Moreira, Margarida Rodrigues, Gustavo Beça

**Affiliations:** 1 Physical and Rehabilitation Medicine, Centro Hospitalar Tondela Viseu, Viseu, PRT; 2 Spinal Cord Injuries Department, Centro de Reabilitação do Norte - Centro Hospitalar de Vila Nova de Gaia Espinho, Porto, PRT; 3 Physical Medicine and Rehabilitation, Centro de Reabilitação do Norte - Centro Hospitalar de Vila Nova de Gaia Espinho, Porto, PRT

**Keywords:** pain perception, analgesic treatment, child disorder, muscle spasticity, botulinum toxins type a

## Abstract

Introduction: Botulinum toxin type A injections are a first-line treatment for spasticity in children. Our purpose is to delineate the national landscape concerning pain management for botulinum toxin type A injections in pediatric patients and to formulate a protocol grounded in current scientific evidence.

Methods: We conducted a nationwide survey targeting physical medicine and rehabilitation specialists performing botulinum toxin type A injections for focal spasticity in children in Portugal. We conducted a literature review to compare the survey results with clinical guidelines, good practice manuals, and protocols published in the literature. Finally, we developed a procedural protocol for pain management in botulinum toxin procedures.

Results: The survey was completed by 17 out of 18 identified specialists. All but one use some form of periprocedural analgesia. Five do not use any type of sedation. The majority do not assess pain during the procedures. From the reviewed articles, we obtained 23 articles, 19 of which provided data for detailed analysis.

Conclusions: A prevailing concern centers around pain management during botulinum toxin procedures in children. Nevertheless, a distinct absence of uniformity persists in appraising and managing procedure-related pain. This notion is further underscored by the marked heterogeneity and paucity of published literature within this realm. The systematic implementation of a procedural protocol thus becomes highly crucial.

## Introduction

Botulinum toxin type A (BoNT-A) injections are a first-line treatment for focal, multifocal, and segmental spasticity in children [1,2]. According to the Royal College of Physicians [3], the goals of BoNT-A intervention are pain control, reduction of involuntary movements, prevention of contractures and deformities, improvement of passive and/or active functions, and enhancement of mobility, including gait. Achieving these goals involves the periodic administration of BoNT-A to maintain the benefit and prevent or delay the development of irreducible contractures. In most cases, the infiltration of several muscles per session is necessary, as most children exhibit spasticity in multiple segments [4].

The guidance method for the injections, as well as the pain management strategy during the procedure are two aspects that should be considered when administering BoNT-A to children [2,4]. There are several guidance methods available, including palpation, anatomical landmarks, electromyography, electrical stimulation, and ultrasound [4]. Among these, ultrasound is a real-time dynamic imaging method with excellent spatial resolution and safety profile, and it has been the preferred method in studies with pediatric populations [4,5].

According to the Portuguese Directorate General of Health [6], invasive procedures are the most frequent cause of pain in children seeking healthcare services, often together with fear and anxiety. These factors worsen the pain, underscoring the importance of minimizing anticipatory anxiety through a comprehensive pain management approach right from the initial interaction with healthcare services [6]. Taking this into consideration, it becomes imperative to formulate and implement a suitable protocol for pain management aimed at mitigating the pain and anxiety linked to BoNT-A injections [2]. This protocol should consider not only analgesia strategies but also pain assessment [6].

Sedation is mostly employed to alleviate pain and anxiety, thus promoting the child's cooperation and ensuring the safe execution of the procedure. The child's capacity to control their behavior and cooperate depends not only on their age but also on their level of cognitive and emotional development [7]. In addition to pharmacological strategies, non-pharmacological methods should be applied, as they have the potential to reduce the requirement for more profound sedation, especially in minor and brief procedures [6,7]. Sedation of pediatric patients involves some risks, which become more pronounced in children with developmental impairment. These children, when exposed to sedative drugs during invasive procedures, exhibit an incidence of hypoxia three times higher compared to children without developmental impairment [8]. Appropriate drug selection, a comprehensive grasp of their pharmacokinetics, pharmacodynamics, and potential interactions, along with the presence of a physician skilled in advanced pediatric life support, collectively assume a vital role [7].

While clinical guidelines and protocols exist for sedation and analgesia in therapeutic or diagnostic procedures [2,6,7,9], the authors have found a gap concerning their application and standardization in procedures involving BoNT-A injections in children. There is a notable scarcity of publications in this field. Furthermore, there is no published data in Portugal regarding the assessment and management of pain from BoNT-A injections in children.

This study aims to outline a procedural protocol for the effective management of pain during BoNT-A injections in pediatric patients. This proposal is grounded in an understanding of the real-world clinical practices across different centers in Portugal, as well as the information outlined in the literature.

## Materials and methods

We conducted a nationwide multicenter survey targeting all physical medicine and rehabilitation (PM&R) specialists practicing in Portugal who use BoNT-A for the treatment of spasticity in pediatric patients. We juxtaposed the obtained results with existing literature and devised a protocol proposal for evaluating and addressing pain during BoNT-A injections in children.

Development of the nationwide multicenter survey

**Figure 1:**
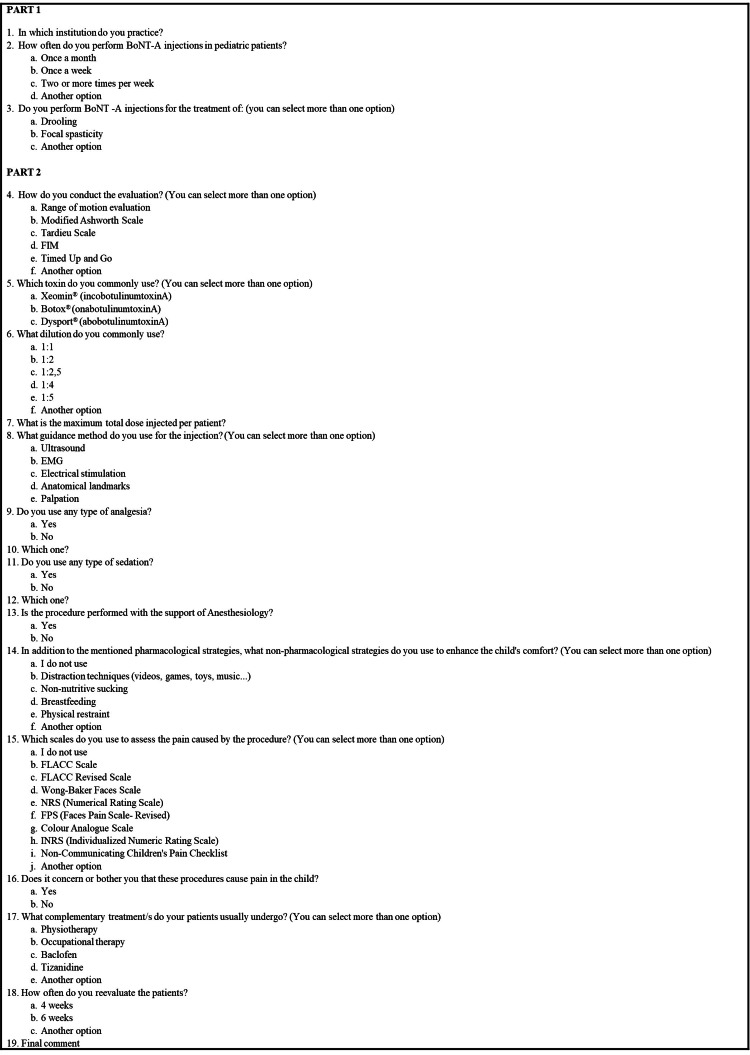
A survey was conducted with PM&R specialist, who currently perform BoNT-A injections in children as part of their clinical practice in Portugal BoNT-A: Botulinum toxin type A; FIM: Functional independence measure; EMG: Electromyography; FLACC: Face, legs, activity, cry, consolability

We designed the survey based on our clinical practice, anchored in clinical guidelines, best practice manuals, and protocols published regarding the use of BoNT-A for the treatment of focal spasticity in children [1,4], alongside considerations for periprocedural pain management [2,6,7,9]. The survey was divided into two parts: an initial section for sociodemographic and clinical characterization of the PM&R specialist and a second section focused on BoNT-A injections in focal spasticity in children (Figure 1).

Participant selection and survey administration

We identified all PM&R specialists working in pediatric rehabilitation in Portugal who currently perform BoNT-A injections in children as part of their clinical practice. Their contact information was obtained through the interest group "Pediatric Rehabilitation Section of the Portuguese Society of PM&R," through which the survey was shared in an online format using the Google Forms® application.

We excluded PM&R specialists who exclusively perform BoNT-A injections in adults. Medical residents, neurologists, pediatricians, and anesthesiologists were also excluded.

We conducted a descriptive analysis of the results using Microsoft Excel® software. Specific cases and/or comments were also subjected to analysis and are presented when deemed relevant.

This study follows the principles of the Declaration of Helsinki. The completion of the survey was considered the participant's consent. The questionnaire was filled out anonymously, and the collected data were treated confidentially.

Literature review

**Figure 2:**
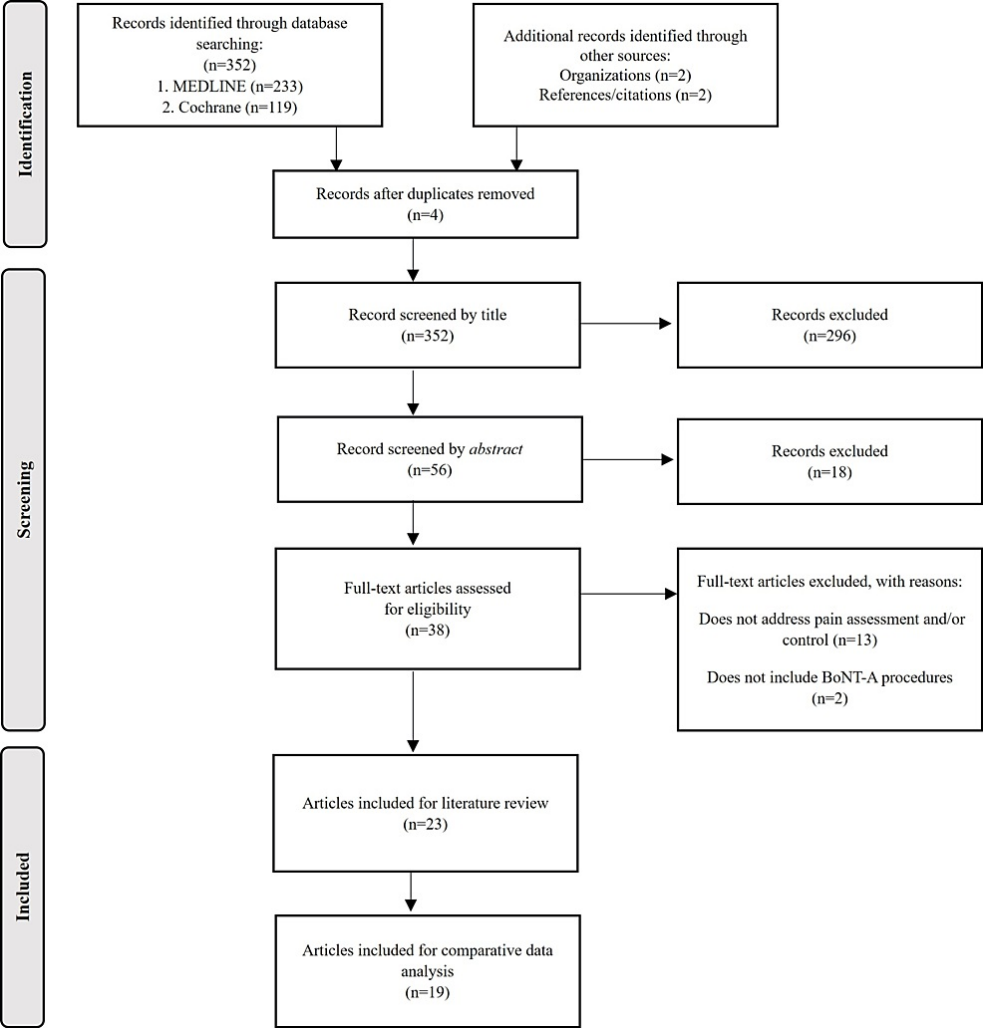
Literature review flowchart.

We conducted a literature review by searching the MEDLINE (PubMed) and Cochrane Library databases. We used the search terms ((botulinum toxin) AND (children)) AND ((best clinical practices) OR (protocol) OR (guidelines)) and ((botulinum toxin) AND (children)) AND ((analgesia) OR (sedation)). Additionally, the reference lists of selected articles were analyzed to identify relevant papers that might not have been obtained in the initial search. Only articles published within the last 15 years were included, with no language-based exclusions. The acquired articles were imported into the Mendeley® reference management software, where duplicate documents were automatically removed. In the initial stage, articles were selected based on their titles; in the second stage, abstracts were read; and in the third and final stage, articles were read in their entirety. Articles that did not address pain assessment and/or management, as well as those not involving BoNT-A injections, were excluded from the final review (Figure 2). The review was conducted in November 2022.

For the obtained articles, we created a form with the most relevant topics to extract: first author, year of publication, study design, main goal, population, sedation and analgesia, adverse effects, non-pharmacological strategies, pain assessment, and main conclusions. We employed this form as a tool for detailed and comparative analysis of the extrapolated data.

## Results

Nationwide multicenter survey

The survey was available for 33 days, from October 12th to November 13th, 2022, and was completed by 17 out of total 18 identified specialists (94%) (Table 1). Out of the 17 responses, two were excluded: one was disregarded because the specialist did not employ BoNT-A in children, and the other because the specialist did not employ BoNT-A for treating focal spasticity. The collected responses showed geographical representation throughout the country, encompassing regions from north to south, including the islands. Of the respondents, 53.3% (n=8) dedicate at least one weekly session (morning or afternoon) of their clinical practice to focal spasticity treatment in children using BoNT-A.

**Table 1:** National multicenter survey results. FIM: Functional independence measure; EMG: Electromyography; EMLA®: Eutectic mixture of local anesthetics (lidocaine + prilocaine)

	n (%)
Identified specialists	18 (100)
Completed surveys	17 (94.4)
Valid surveys for analysis	15 (88.2)
Excluded responses	2 (11.8)
Geographical distribution	
North	7 (46.6)
Center	5 (33.3)
South	2 (13.3)
Islands	1 (6.7)
Frequency of procedures	
≥1 per week	8 (53.3)
≥1 per month	7 (46.7)
Focal spasticity assessment	
Modified Ashworth Scale	15 (100)
Range of motion	13 (86.7)
Tardieu scale	5 (33.3)
FIM	3 (20)
Timed up and go	2 (13.3)
Goal Attainment Scaling	2 (13.3)
10-meter walk test	2 (13.3)
Botulinum Toxin type A	
Botox^®^ (onabotulinumtoxinA)	15 (100)
Dysport^®^ (abobotulinumtoxinA)	14 (93.3)
Xeomin^® ^(incobotulinumtoxinA)	4 (26.7)
Guidance method	
Ultrasound	9 (60)
Anatomic landmarks/Palpation	6 (40)
EMG/Electrical stimulation	0 (0)
Analgesia	
Yes	14 (93.3)
No	1 (6.7)
Analgesic drugs	
EMLA^®^	10 (71.4)
Ethyl chloride	4 (28.6)
Paracetamol	2 (14.3)
Nitrous oxide	2 (14.3)
Nitrous oxide + EMLA^®^	1 (7.1)
Sedation	
Yes	10 (66.7)
No	5 (33.3)
Sedative drugs	
Nitrous oxide	4 (40)
Sevoflurane	3 (30)
Midazolam	2 (20)
Propofol	1 (10)
Nitrous oxide + midazolam	1 (10)
Unspecified general anesthesia	2 (20)
Anesthesiology support	
Yes	7 (70)
No	3 (30)
Non-pharmacological strategies	
Distraction techniques (videos, games, toys, music...)	14 (93.3)
Physical restraint	3 (20)
Non-nutritive sucking	1 (6.7)
Breastfeeding	1 (6.7)
Pain assessment	
Yes	4 (26.7)
No	11 (73.3)
Pain assessment scales	
Numerical Rating Scale (NRS)	4 (100)
Visual Analog Scale (VAS)	2 (50)
Faces Scale	1 (25)
FLACC (Face, Legs, Activity, Cry, Consolability)	1 (25)
Checklist for Non-Communicating Children's Pain Scale (CHEOPS) Revised	1 (25)
Complementary Therapies	
Physiotherapy	15 (100)
Occupational therapy	15 (100)
Baclofen	15 (100)
Tizanidine	6 (40)
Orthoses	3 (20)
Serial casting	3 (20)
Reassessment after the procedure	
6 weeks	11 (73.3)
4 weeks	2 (13.3)
3 months	1 (6.7)
4 to 6 months	1 (6.7)

All specialists (n=15) employ the Modified Ashworth Scale for spasticity assessment. Additionally, 86.7% (n=13) evaluate joint ranges of motion, and 33.3% (n=5) complement their assessment with the Tardieu scale. Other methods for functional assessment include the Functional Independence Measure (20%, n=3), Timed Up and Go (13.3%, n=2), Goal Attainment Scaling (13.3%, n=2), and the 10-meter walk test (13.3%, n=2). All specialists (n=15) use onabotulinumtoxinA, 93.3% (n=14) use abobotulinumtoxinA, and 26.7% (n=4) use incobotulinumtoxinA. Among them, 6.7% (n=1) solely use onabotulinumtoxinA, 66.7% (n=10) use two toxins (onabotulinumtoxinA and abobotulinumtoxinA), and 26.7% (n=4) use all three toxins (onabotulinumtoxinA, abobotulinumtoxinA, and incobotulinumtoxinA). The dilution and maximum dosage utilized are contingent upon the chosen toxin for the procedure. Sixty percent (n=9) use ultrasound as a guidance method, while the remaining 40% (n=6) use anatomical references and/or palpation.

Regarding periprocedural analgesia, 93.3% (n=14) use some form of analgesia. Ten of them (71.4%) apply lidocaine and prilocaine cream (EMLA®), 28.6% (n=4) employ ethyl chloride, 14.3% (n=2) use paracetamol, and 14.3% (n=2) employ nitrous oxide, which, in one case (7.1%), is used in combination with EMLA®.

Regarding sedation, 33.3% (n=5) do not employ any form of sedation for BoNT-A injection. Among the 66.7% who do use sedation (n=10), 40% (n=4) use nitrous oxide, 30% (n=3) use sevoflurane, 20% (n=2) use midazolam, 10% (n=1) use propofol, and 20% (n=2) use resort to some form of general anesthesia without specifying which one. One specialist uses a combination of nitrous oxide and midazolam. Seventy percent (n=7) perform the procedure with the assistance of an anesthesiologist. Distraction techniques such as videos, games, toys, and music are used in 93.3% (n=14) of cases. Other non-pharmacological strategies include physical restraint (20%, n=3), non-nutritive sucking (6.7%, n=1), and breastfeeding (6.7%, n=1).

Eleven specialists (73.3%) do not use scales to evaluate the pain caused by the procedure. Among those who do use them (26.7%, n=4), they employ more than one scale, with the Numeric Rating Scale (NRS) being used by all (100%, n=4). The Visual Analog Scale (VAS) (50%, n=2), the Faces Pain Scale (25%, n=1), the FLACC (Face, Legs, Activity, Cry, Consolability) scale (25%, n=1), and the Non-Communicating Children's Pain Checklist (25%, n=1) are also used. All respondents (n=15) are concerned regarding the discomfort caused to children by these procedures.

All participants (n=15) point out physiotherapy, occupational therapy, and pharmacological treatment with baclofen as the complementary therapies commonly performed for their patients. Other complementary therapies included tizanidine (40%, n=6), orthoses (20%, n=3), and serial casting (20%, n=3). Eleven specialists (73.3%) reevaluate their patients at six weeks; 13.3% (n=2) reevaluate at four weeks; 6.7% (n=1) at three months; and 6.7% (n=1) between 4 and 6 months.

Literature review

From the conducted research, we obtained 23 articles for review, the majority of which were published in journals ranked in the first or second quartiles (78%, n=18) in their respective years of publication. Out of the 23 articles, 12 (52%) were published in the last four years (since 2018), and the majority (n=21) were written in English. We obtained four randomized controlled trials, eight prospective studies, seven retrospective studies, one cross-sectional questionnaire, one literature review, one clinical guideline, and one commentary. Due to the significant heterogeneity among the articles, we could not conduct a meta-analysis. However, it was possible to extrapolate data for analysis and comparison among 19 out of the 23 articles obtained (Table 2).

**Table 2:** Summary of the data extracted from a comprehensive analysis of the articles obtained through the literature review. na: not applicable/omitted; BoNT-A: Botulinum Toxin type A; n: number; x: average; sd: standard deviation; CP: Cerebral palsy; GMFCS: Gross motor function classification system; GA: general anesthesia; TBI: Traumatic brain injury; BART: Biofeedback assisted relaxation training; FLACC: Face, legs, activity, cry, consolability; FPS: Faces pain scale; VAS: Visual analog scale; pts: points; IV: intravenous; IM: intramuscular; VRHMD: Virtual reality head-mounted display; EMLA®: Eutectic mixture of local anesthetics (lidocaine + prilocaine); CHEOPS:  Children's hospital of Eastern Ontario pain scale;  NRS: Numerical rating scale; FPS-R: Faces pain scale- revised

First author	Year	Design	Main goal	Population	Sedation and/or analgesia	Adverse effects	Non-pharmacological strategies	Pain assessment	Main conclusions
Portuguese Directorate-General of Health [6]	2012	Guidelines	Provide technical guidelines on pain control during invasive procedures in children.	Children aged 1 month to 18 years, without further specification.	Recommended to be used systematically, appropriate to each situation.	na	Recommended to be used systematically, appropriate to each situation.	In all situations: assess the pain before, during, and after the procedure.	Use systemic analgesia and conscious sedation systematically in BoNT-A injections, with the latter being optional if the child is cooperative.
Gubbay [16]	2009	Letter to the editor	Evaluation of the safety and efficacy of midazolam, ketamine and intranasal fentanyl in children undergoing BoNT-A injection.	1. n=123 (age x=7,08 (sd 3,92)); children with cerebral palsy (CP) (no further specification) 2. n= 108 (age x=8,92 (sd 3,92)); children with CP (GMFCS I n=63, II n=30, III n=15; Majority with spastic diplegia without significant involvement of the upper limbs.	1. Oral midazolam 0.5mg⁄kg + anesthetic cream / midazolam 0.5mg⁄kg + oral ketamine 3mg⁄kg + anesthetic cream; 2. Intranasal fentanyl 1.5mcg/kg + anesthetic cream	1. Transient distress post-procedure (1) 2. No serious adverse effects reported.	Distraction techniques.	Assessment of pain tolerance and level of parental satisfaction with sedation (method not specified).	In departments where access to nitrous oxide and local anesthetic is limited, these are safe and effective alternatives. Authors are increasingly using intranasal fentanyl with anesthetic cream as a replacement for midazolam, as it provides less sedation, better analgesia, easier administration, and faster recovery. Midazolam plus ketamine if deeper sedation is required.
Friedrichsdorf [17]	2022	Comment	Comment on best practices in the administration of intramuscular BoNT-A to children.	na	na	na	na	na	The best clinical practice to prevent pain and anxiety in children undergoing intramuscular BoNT-A currently involves topical anesthesia, nitrous oxide, and integrative non-pharmacological strategies for all children, always. Failing to do so in developed countries is unacceptable.
Ostojic [18]	2022	RCT	To compare biofeedback assisted relaxation training (BART) with distraction therapy for pain during BoNT-A treatment	n=38 (age x=13,42 (7-18); children with CP, traumatic brain injury (TBI) or hereditary spastic paraplegia.	No	na	Biofeedback assisted relaxation training (BART) and distraction therapy	FLACC, Faces Pain Scale - Revised, Children's Fear Scale, Numerical Rating Scale: anxiety e State-Trait Anxiety Inventory	Children reported similar pain when using BART and distraction therapy. Those who used BART before distraction therapy reported less pain and anxiety during treatments.
Kumar [12]	2009	Letter to the editor	Adding topics to the discussion of Zier et al 2008 study; comparing 50% nitrous oxide/O2 mixture (Entonox^®^) with continuous 70% nitrous oxide/O2 mixture.	n=33 (age x= 8,08 (1,92-17,42)); children with CP (GMFCS I n=11, II n=6, III=6, IV=5, V=5; hemiplegia=8, diplegia=11, quadriplegia= 7, another=7)	1. 50% nitrous oxide/O2 mixture (Entonox^®^); 2. Midazolam + topical analgesia	No adverse effects	na	FLACC, FPS, VAS, Parent satisfaction (likert 3 pts)	For older children (>5 years old) who can comply with verbal instructions and require a limited number of injections, self-administered Entonox^®^ can provide a similar level of procedural pain control to the continuous flow nitrous oxide ⁄oxygen system used by Zier et al. It has other advantages in terms of potentially fewer adverse effects and lower resource overheads.
Soudant [19]	2013	Letter to the editor	na	Children with CP	General anesthesia (GA)	na	na	na	They recommend the use of GA to optimize BoNT-A treatment in patients with CP. This approach facilitates the assessment of contractures and enables the use of electrostimulation.
Forrester [15]	2012	Prospective study	Comparing carer satisfaction and perception of procedure related pain and distress for intramuscular BoNT-A injections administered to children and adolescents with CP using 2 methods of analgesia / sedation: conscious sedation using nitrous oxide and topical local anesthetic versus general anesthesia	n=171 (age x=7,08); children with CP: conscious sedation group (1) with homogeneous distribution across the 5 levels of GMFCS.; GA group (2) with a predominance of GMFCS II and III.	1. Nitrous oxide + topical local anesthetic; 2. GA	No serious adverse effects	na	A specific carer completed assessment tool utilizing Likert scales, for pain perception and satisfaction about the treatment	The perception of distress by caregivers and physicians during entry into the procedure room and mask placement was similar with conscious sedation and GA. The pain perceived by caregivers caused by the procedure under conscious sedation was significantly greater than under GA. There was no significant difference in overall satisfaction with either modality.
Zier [11]	2008	RCT	Comparing the efficacy of sedation with enteral midazolam to inhaled nitric oxide in children undergoing BoNT-A injections for spasticity.	n=50 (age x= 8,17 (1-16)); children with CP: group 1 GMFCS I n=0, II n=13, III=3, IV=6, V=3; hemiplegia=8, diplegia=5, triplegia=0, quadriplegia=9, another=3; group 2 GMFCS I n=4, II n=11, III=1, IV=7, V=2; hemiplegia=9, diplegia=6, triplegia=3, quadriplegia=7, another=0 (No statistical differences between groups)	1. Enteral midazolam entérico (0.35 a 0.5mg/kg); 2. 70% inhaled nitric oxide	Midazolam: hypoxemia (1); Inhaled nitric oxide: nausea (1), vomiting (4), headache and pallor (1), hypoxemia (3)	Distraction technics (storytelling, soothing discourse)	FLACC scale, Perception of ease of the procedure by the doctor and nurses (0-10), level of satisfaction of parents with the comfort of their children during the procedure (0-10), comparing the level of sedation with previous procedures (5-point Likert scale).	Comparable levels of sedation between nitric oxide and midazolam, but those who received nitric oxide were less sedated at the end of the procedure; nitric oxide more effective in reducing pain than midazolam; parents expressed satisfaction with both types of sedation, but those in the nitric oxide group reported better sedation compared to previous procedures; nitric oxide appears to be an effective sedation method for children undergoing BoNT-A infiltration, but further studies are needed.
Chow [20]	2016	Retrospective study	Describing the authors' experience with a ketamine-based sedoanalgesia protocol in terms of efficacy and safety for children undergoing BoNT-A injections.	n=87 (age x=5,42 (1,42-13,17)): ketamine + midazolam (123); ketamine (29); children with CP: GMFCS II n=52, ≥III n=35	Topical anesthetic cream; Intravenous ketamine 1.0 mg/kg and intravenous midazolam 0.1 mg/kg	Rashes, (4); náusea and vomiting (3); limb tremors (1); mild headache (1); nightmares on the evening of the procedure (1)	na	Not conducted.	Ketamine-based sedoanalgesia in BoNT-A injections in children is safe and effective.
Bakheit [21]	2003	Retrospective study	Analyzing the pattern of clinical practice in relation to the use of BoNT-A in the management of muscle spasticity in children.	n=758 (age x=9,04); children with CP (94%), Stroke, Hereditary spastic paraplegia and Brain tumor; High prevalence of quadriplegia and intellectual impairment.	GA; Local anesthetic and/or midazolam	na	na	na	Results from 17 centers: none in 6; GA in 4; local anesthetic and/or midazolam in 7. The study shows that infiltrations can be performed without GA in most cases.
Louer [22]	2019	Retrospective study	Investigate the safety and efficacy of a propofol and ketamine-based sedation protocol in pediatric patients with CP receiving BoNT-A injections.	n=164 (age x=9 (4-11)); children with CP: GMFCS I n=5, II n=21, III=50, IV=55, V=33	Initial bolus of ketamine 0.5 mg/kg IV + initial bolus of propofol 2 mg/kg IV, if movement, additional boluses of 0.5-1 mg/kg of propofol until desired sedation is achieved.	Hypoxemia (9,6%); Transient apnea (1,4%); Hypoxemia + apnea (0,9%); not serious	na	na	The study demonstrated that the propofol + ketamine protocol was entirely safe and effective. This combination allows for faster recovery times. Due to the high incidence of adverse effects in this population, these patients require surveillance and monitoring provided by physicians trained in pediatric airway management and cardiorespiratory monitoring.
Patel [23]	2018	RCT	Demonstrate the efficacy of using commercially available virtual reality head-mounted display (VRHMD) for virtual reality analgesia (VRA) in children undergoing BoNT-A injections for spasticity control. Further, to see the psychological benefits of VRA for pediatric patients and caregivers versus normal practice, and to reduce the number of children requiring general sedation for spasticity management.	n=20 (age 6-16); without further specifications	na	na	Google Cardboard Virtual reality head mounted display powered by an iPod touch; Oculus Rift	FLACC before, during, and after the procedure, by an observer and parents.	Patients randomized to virtual reality analgesia showed a significant improvement in their ability to tolerate pain and discomfort during BoNT-A injections compared to patients in the control group.
Sozbilen [24]	2022	Retrospective study	Compare functional outcomes and types of anesthesia administered in patients who received repeated BoNT-A injections; determine the most appropriate anesthesia type for multiple BoNT-A injections.	n=75 (age x=3,79 (1,58-12,25)); children with CP: GMFCS I n=0, II n=11, III=34, IV=27, V=3	1. Midazolam + ketamine; 2. Sevoflurane via anesthetic mask; 3. Sevoflurane via laryngeal mask	Bronchospasm (2) and vomiting (8)		Evaluated the level of sedation using the Modified Aldrete Recovery Scoring System.	Sedoanalgesia with Midazolam + ketamine promotes faster recovery than mask anesthesia or laryngeal mask anesthesia in the first 3 applications. There was no difference between the 3 types of anesthesia in 4 or more repeated applications. In repeated treatments with sedoanalgesia and other types of anesthesia, longer recovery periods have been observed.
Mian [25]	2021	Retrospective study	Evaluate if intramuscular midazolam is useful in reducing injection-related anxiety during "in-clinic" BoNT-A procedures.	n=71 (age x=12 (0-25)); children with CP n=61 (GMFCS II-V), other n=10 (mitochondrial diseases, TBI…)	Topical anesthetic cream + Midazolam (adjusted for weight) or GA.	No adverse effects reported.	na	Assessment of sedation and "breakthrough crying."	The administration of intramuscular midazolam for spasticity treatment is effective for sedation and without adverse effects. Therefore, it is a feasible approach for reducing anxiety in the pediatric population undergoing injections.
O'Flaherty [26]	2021	Cross-sectional survey	To describe current rehabilitation pediatricians’ use of intramuscular BoNT-A to manage hypertonicity.	na	Sedation used by 97%; midazolam 94%; local anesthetic cream 84%; inhaled nitric oxide 84%; GA 84%; propofol 7%.	na	CoolSense Medical^®^ and Buzzy XL Personal^®^ devices; therapist; assistant	na	Consistency in maximum doses used (OnabotulinumtoxinA) and widespread use of sedation for BoNT-A administration.
Brochard [13]	2009	Prospective study	Evaluate the effectiveness of an analgesic protocol using nitrous oxide and EMLA^®^ in children undergoing BoNT-A injections.	n=34 (age x=5,94 (2-15)); children with CP n=33 (hemiplegia n=10, diplegia n=12, tetraplegia n=11), adductor hypertonia n=1	Nitrous oxide and EMLA^®^	Vomiting (2) and vivid dreams (1)	Distraction techniques (storytelling, music, or other methods according to the child's preference)	Children’s Hospital of Eastern Ontario Pain Scale (CHEOPS), VAS (>6 years), FPS (<6 years), VAS to parents (if not possible to ask the child or <4 years)	The combination of nitric oxide + EMLA^®^ is effective in only 50% of the children; the puncture phase is much less painful than the localization and injection phases. Further studies are needed to define a better protocol.
Brochard [27]	2011	Prospective study	To determine technical and clinical factors associated with pain when using na analgesic protocol with 50% nitrous oxide/oxygen and anesthetic cream (EMLA^®^) for children with CP undergoing BoNT-A injections.	n=50 (age x=6,6 (1-18)); children with CP: GMFCS I n=10, II n=20, III=6, IV=10, V=4	50% Nitrous oxide/O2 + EMLA^®^	Vomiting, nausea, and vivid dreams	Distraction techniques (storytelling, music, or other methods according to the child's preference)	CHEOPS	Clinical characteristics do not strongly correlate with the success or failure of the 50% nitric oxide / EMLA^®^ protocol, and this protocol does not equally prevent all phases of TB-A infiltration. Further studies are needed on products and dilutions to help reduce pain.
Gambart [14]	2007	Prospective study	To evaluate the efficiency and tolerance of analgesic treatment with nitrous oxide and topical eutectic mixture of local anesthetics (EMLA^®^) in children undergoing BoNT-A injections for focal spasticity.	n=40 (age 2-17); children with CP n=38 (diplegia n=12, hemiplegia n=9, triplegia n=1, tetraplegia n=7, cognitive impairment n=10), Stroke n=1, Myelomeningocele n=1	50% Nitrous oxide/O2 (Entonox^®^) + EMLA^®^	Visual hallucinations and euphoria (3)	na	Likert scale (3 points) for child's movements/ expressions	Analgesia with nitric oxide + EMLA^®^ was effective but insufficient in half of the children, requiring protocol modifications; Precise pain assessment in children with major cognitive deficits is challenging; Clinical manifestations are related to pain but also to anxiety and stress.
Fisher [28]	2018	Retrospective study	Identify factors that can be targeted to reduce periprocedural pain with BoNT-A injections.	n=249 (age x=9,2); children with CP n=189, other neurological diseases without further specifications n=60	Vapo-coolant spray and topical anesthetics.	na	Child Life specialists	Cognitively capable: NRS (during and 5 minutes after); if not: parental assessment at the same timings.	Pain reported as absent or mild after the procedure in at least 95% of cases; younger age, use of topical anesthetics, and injection site related to a significant increase in acute pain during the procedure; acute pain during the procedure and older age related to the presence of pain 5 minutes after the procedure.
Cantador-Hornero [2]	2019	Transversal study	To evaluate the impact of the sedation-analgesia technique on the pain experienced by the patient with CP during BoNT-A injections.	n=124 (age x=6,75 (2-16)); children with CP: GMFCS I n=32, II n=33, III n=29, IV n=30	4 groups: 1. without sedoanalgesia or topical anesthetic cream; 2. inhaled nitric oxide; 3. deep IV sedation; 4. oral or rectal benzodiazepine.	na	na	<3 years: FLACC scale by trained observer; 3-7 years: Wong-Baker Faces Scale and VAS; ≥ 8 years: Walco and Howite VAS; Parental satisfaction: 5-point Likert scale; Healthcare professional satisfaction with sedation: 5-point Likert scale.	Pain was significantly lower in patients who received deep IV sedation compared to the others; however, general anesthesia may not be recommended for all children with CP. The authors emphasize the importance of appropriate protocol selection for sedoanalgesia based on each child's condition.
León-Valenzuela [10]	2021	Retrospective study	To describe the author’s experience with a protocol based on sevoflurane sedation to control pain and agitation during BoNT-A infiltration in children with CP, especially in terms of safety and efficacy.	n=74 (age x=7 (1-17)); children with CP: GMFCS I n=2, II n=36, III n=12, IV n=14, V n=10 (hemiplegia n=31, paraplegia n=14, tetraplegia n=29)	Sevoflurane	Nausea and vomiting (3,88%) and hypoxemia (2,07%); No serious adverse events.	na	Not conducted; healthcare professionals' satisfaction.	Sedation with sevoflurane demonstrated promising results in terms of safety and efficacy for pain and agitation control during BoNT-A infiltration in daily clinical practice. Furthermore, it can facilitate infiltration by allowing assessment under sedation and multi-level injections with good tolerance.
Frenn [29]	2019	RCT	Study the application of Virtual Reality in BoNT-A for the treatment of spasticity.	n=10 (age x=14,67); children with CP and other unspecified diseases.	na	na	na	Self-report, parental and medical information: FPS-R, VAS	Potential of Virtual Reality to decrease the perception of pain and anxiety during painful procedures in individuals with spasticity associated with developmental disorders.
Nugud [9]	2021	Literature review	Identify the different procedural sedation and analgesia agents used for BoNT-A injections in children with CP to assess their feasibility and adverse effects.	Children with CP, without further specifications.	More frequent: inhaled nitric oxide, EMLA^®^, and midazolam (administered through different routes); IV ketamine; Remifentanil and propofol.	No serious adverse events.	Distraction techniques (clowns)	Level of parental satisfaction (but not clear)	The combination of inhaled nitrous oxide with EMLA^®^ cream showed promising primary results. However, ketamine and midazolam combination could be a safe alternative. Currently, there is no sufficient data to draw on the superiority of any modality. Further high-quality studies are warranted.

The sample sizes were very different among the studies, with variability ranging from n=10 to n=758 and an average sample size of 120 children with a mean age of 8.36 years old (min. 3.79; max. 14.67). In 19 studies, the included children had cerebral palsy (CP), while in the remaining four studies, no information was provided regarding the underlying pathology. Among these 19 studies, 10 characterized their samples according to the Gross Motor Function Classification System (GMFCS), showing a balanced distribution across levels I-IV (level I/II x=36, level III/IV x=35) and a lower frequency for level V (x=7). Six out of the 23 studies (26.1%) included other pathologies such as stroke, traumatic brain injury, and mitochondrial diseases.

Among the studies that investigated pharmacological strategies for pain control during BoNT-A injections in children (n=15), the most studied drugs were midazolam (n=8), nitrous oxide (n=7), ketamine (n=4), and sevoflurane (n=2). A topical anesthetic was used in combination with other drugs in eight out of the 15 studies. Of these 15 studies, all except one [10] analyzed the efficacy of nitrous oxide prospectively. When compared to midazolam, nitrous oxide appeared to be more effective and allowed for quicker recovery [11], and it was considered a better option when used in cooperative children without comprehension impairment [12]. Nitrous oxide, in combination with EMLA®, was effective in only half of the children in the studies by Brochard et al. and Gambart et al. [13,14]. Forrester et al. concluded that conscious sedation with nitrous oxide in combination with a local anesthetic could be a viable alternative to general anesthesia [15]. On the other hand, Cantador-Hornero et al. found that patients who received deep intravenous sedation experienced significantly less pain compared to others (absence/topical anesthetic cream, nitrous oxide, and oral or enteral benzodiazepine) [2]. León-Valenzuela et al. retrospectively studied the efficacy of sevoflurane and concluded that a sedation protocol with sevoflurane resulted in a high effectiveness rate in pain control during BoNT-A injections, being successful in 100% of cases [10].

Regarding adverse effects, in the studies where they were listed (n=13), all authors acknowledged non-serious and transient adverse effects when they occurred. The most frequently described adverse effects were nausea, vomiting, and hypoxemia. Concerning hypoxemia, it was reported in all studies as transient and immediately resolved with oxygen therapy. Zier et al. reported oxygen levels below 92%, which were promptly and incident-freely resolved by administering 100% oxygen [11].

The non-pharmacological strategies were employed in combination with sedative and analgesic drugs in five of the studies, with distraction techniques being the most reported. Four of the selected studies examined the impact of non-pharmacological strategies on periprocedural pain control, specifically virtual reality (n=2), biofeedback-assisted relaxation training (n=1), and the presence of child life specialists (n=1). All of them reported positive outcomes in terms of alleviating pain and anxiety.

In most studies (n=14), pain and/or anxiety related to the procedure were assessed. The most used pain assessment scales were the FLACC scale (n=5), the VAS (n=5), and the Faces Pain Scale/Faces Pain Scale Revised (n=5). Likert-type scales for evaluating satisfaction and/or pain perception from parents or healthcare professionals were also used (n=5). 

## Discussion

The results obtained from conducted survey demonstrate a clear concern among specialists about managing the pain caused by BoNT-A injections in pediatric patients. However, there is a noticeable lack of standardization in both pain control and assessment during the procedure. Similarly, the significant heterogeneity and limited number of published studies on the topic reinforce this notion and restrict the establishment of measures to optimize clinical practice. The utilization of analgesia strategies appears to be unanimous and already widely implemented. However, the absence of a formal pain assessment during the procedure prevents conclusive insight into the effectiveness of the employed strategies. Moreover, procedures of this nature induce anxiety and fear in children, which could contribute to procedural failure and elevate their risk. Hence, sedation, by mitigating these factors, assumes significant importance. As observed, a noticeable gap exists in this regard as well, with one-third of Portuguese PM&R specialists not using any sedative agents. When we compare the questionnaire results with the findings of the conducted research, it becomes evident that the most frequently used drugs by Portuguese PM&R specialists are also those frequently mentioned in the literature, notably nitrous oxide, sevoflurane, and midazolam. Similarly, there is concurrence in the pain assessment scales employed, particularly the FLACC scale, VAS, and Faces Pain Scale/Faces Pain Scale Revised.

Most studies included children with cerebral palsy or other neurological conditions, and they concluded in favor of the efficacy and safety of the studied drugs, describing only non-serious and transient adverse effects.

Guidelines for analgesia and sedation in children undergoing procedures for therapeutic or diagnostic purposes are available [6,7], which serve as a guide for more specific procedures, such as BoNT-A injections. However, these guidelines should be adapted, considering the particularities of these children and the procedure itself.

The medical team should include a PM&R specialist responsible for performing the procedure, an anesthesiologist responsible for managing analgesia and sedation during the procedure, and a nurse, preferably with experience with pediatric patients [7]. The presence of an anesthesiologist adds technical and scientific quality to the team while allowing the physician responsible for administering BoNT-A to focus solely on the procedure.

The chosen place for its execution will depend on the available resources and, simultaneously, will influence the pharmacological and non-pharmacological strategies to be adopted. Options such as the consultation office, treatment room, or operating room are all valid choices. However, it is imperative that these facilities are outfitted with all the essential supplies commensurate with the level of sedation and the drugs intended for use. This includes access to an oxygen supply, an exhaust or gas scavenging system, vital sign monitoring, and a pediatric emergency cart/kit [7].

The choice of pharmacological strategies for pain management will be contingent on various factors, including the targeted sedation level, the child's age and cooperation capacity, and the available material and human resources.

The literature appears to be unanimous in advocating the use of topical analgesia at the intended sites for BoNT-A injections. The most studied strategy involves the combination of lidocaine and prilocaine (EMLA®), to be applied 45 to 60 minutes before the procedure. This local analgesia should be complemented by systemic sedatives and/or analgesic drugs. When targeting minimal or moderate sedation ("conscious sedation"), especially in older children who are cooperative and have no comprehension impairment, midazolam and inhaled nitrous oxide are safe and effective options. Between these two, inhaled nitrous oxide should be preferred over midazolam in cooperative children due to its faster onset and recovery time. However, some studies have shown only 50% effectiveness with the combination of nitrous oxide and EMLA® in children undergoing BoNT-A injections, suggesting that the addition of another analgesic drug might be necessary [13,14]. Children under four years might not tolerate inhaled nitrous oxide, making midazolam a preferable option in these cases. The combination of midazolam and ketamine seems to enable the reduction of the required dosage for both drugs to attain the intended sedative and analgesic outcomes, all while maintaining a high level of safety [16]. For deep sedation, particularly in younger and/or uncooperative children, propofol and ketamine alone or in combination, as well as sevoflurane, could be the best choices. When using ketamine, the prophylactic administration of ondansetron may reduce the occurrence of post-procedural vomiting. The combination of propofol with ketamine appears to be effective and associated with a lower risk of vomiting and hypotension compared to using ketamine alone [22]. Sevoflurane has demonstrated safety and effectiveness, with the advantage of being administered through inhalation and exhibiting good tolerance. It is a suitable choice for performing multi-level injections, as it allows for exploration under sedation [10].

Considering the lack of literature data on preferred drug administration routes, opting for less invasive routes of administration over intravenous and intramuscular methods appears reasonable.

Non-pharmacological strategies should be systematically used and selected according to the child's age and developmental level. For infants, breastfeeding and non-nutritive sucking are recommended. In younger children or those with comprehension impairment, 30% glucose or 24% sucrose solutions, local massage, and distraction techniques such as videos, games, stories, or books are the most suitable and effective strategies. For older children without language or cognitive impairments, prioritizing dialogue and explaining the procedure to them is important. Strategies like positive reinforcement, behavioral modeling, guided imagery or hypnoanalgesia, and muscle relaxation and/or breathing exercises before and after the procedure are appropriate [6]. Other strategies, such as virtual reality, biofeedback-assisted relaxation training, and the presence of Child Life specialists in the room, have been studied and have shown increasing effectiveness in pain control during procedures [18,20,23,29].

Maintaining pain intensity below 3/10 (mild pain) is considered a criterion for high-quality pain control [30]. Therefore, it is imperative to integrate pain assessment scales tailored to the child's age and developmental stage into the procedural protocol. If the child is under four years old or unable to verbalize, the FLACC scale should be used. For children aged 4 to 6, the Faces Pain Scale and Faces Pain Scale- Revised are more appropriate. For those over six years old, the suitable scales are VAS and NRS. For children with multiple disabilities, the FLACC-R scale is recommended [30]. Given the absence of established optimal timing for pain assessment in existing literature, the authors propose the following approach to evaluating pain: utilizing behavior-based scales during the procedure and employing self-report scales immediately afterward.

Vital signs should be continuously monitored throughout the entire procedure [7]. For any procedure requiring sedation, proper preparation is indicated. This includes an appropriate fasting period (2 hours for liquids, 4 hours for breast milk, and 6 hours for light meals), a medical evaluation with a focus on the airway, the American Society of Anesthesiologists (ASA) classification, and a review of anesthesia history [7]. Before the procedure, informed consent should be provided to parents or responsible caregivers for their full reading and authorization of the procedure.

**Figure 3:**
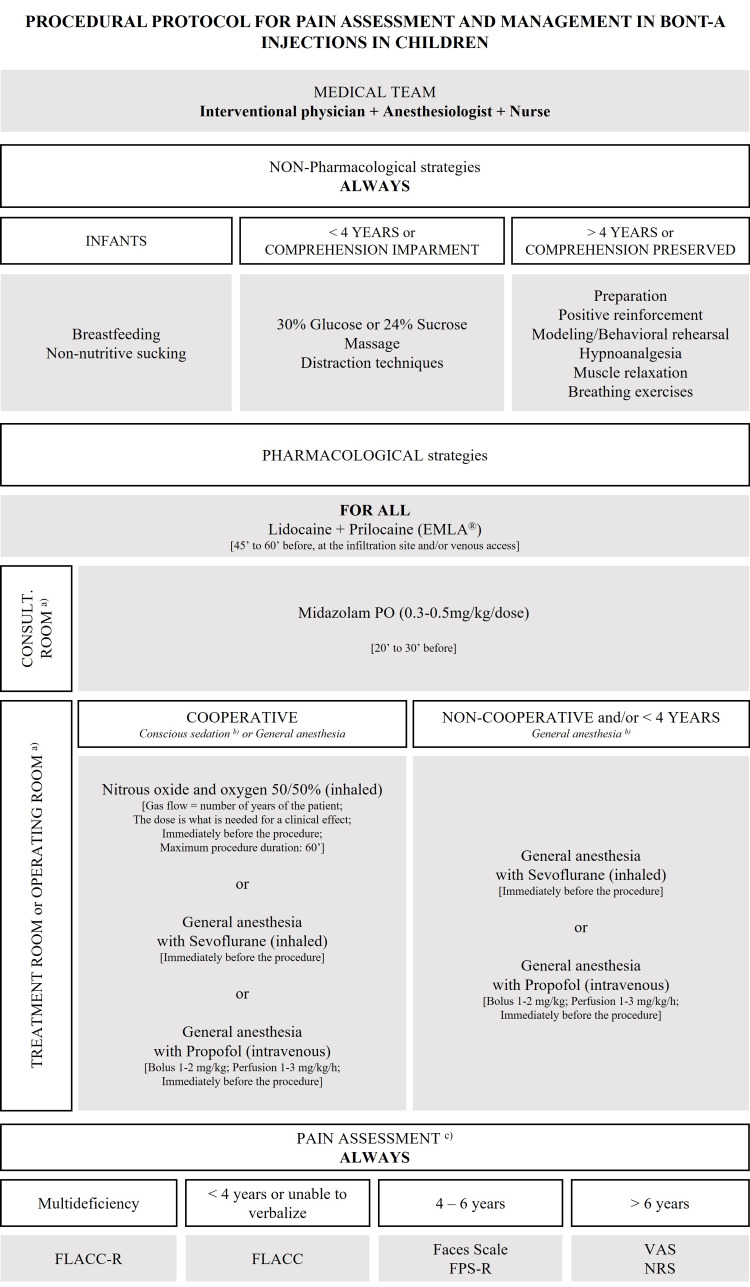
Procedural protocol for pain assessment and management in BoNT-A injections in children. Consult. Room: Consultation room; PO: per os; FLACC-R: Face, legs, activity, cry, consolability–revised; FLACC: Face, legs, activity, cry, consolability; FPS-R: Face pain scale–revised; VAS: Visual analog scale; NRS: Numerical rating scale. a) Equipped with oxygen supply, vital sign monitor, and emergency cart/kit; b) Preferred approach; c) During and/or immediately after the procedure

Based on the results and premises presented, the authors propose a pain control protocol (Figure 3).

According to the authors' knowledge and up to the current date, this is the first study aiming to propose a procedural protocol not only for pain management in BoNT-A injections in children but also for its systematic assessment. This is based on the characterization of current national clinical practice and the analysis of published evidence. The application of a non-validated survey designed for a very specific purpose and directed only at PM&R specialists, as well as the restriction of search criteria to papers published within a predetermined period that specifically refer to pain control in BoNT-A procedures, are limitations of this study.

## Conclusions

The management and assessment of peri-procedural pain in the pediatric population are considered criteria of good clinical practice. In Portugal, data reveals a lack of standardization regarding peri-procedural pain control and assessment despite unanimous concern about this issue. Additionally, in the literature, there is a predominance of heterogeneity among studies in this field. The systematic organization of pharmacological and non-pharmacological strategies, as well as suitable assessment methods for each age group, through a procedural protocol, facilitates their implementation. With this paper, the authors believe they can contribute to improving clinical practice concerning the assessment and management of peri-procedural pain in children undergoing BoNT-A injections.
